# Mechanical ventilation: does the moment of initial use or length of stay on mechanical ventilation have any relation to severity of illness and outcome?

**DOI:** 10.1186/cc14701

**Published:** 2015-09-28

**Authors:** Marina Parente Albuquerque, Eduardo Queiroz da Cunha, Carlos Augusto Ramos Feijó, Allison Emidio Pinheiro Pereira Borges, Natália Linhares Ponte Aragão, Túlio Sugette de Aguiar, Francisco Albano de Meneses

**Affiliations:** 1 Centro de Terapia Intensiva, Hospital Geral de Fortaleza, Fortaleza, CE, Brasil

## Introduction

In the modern ICU, technologies are able to keep patients alive for prolonged periods of time, even despite ongoing life-threatening illness. Mechanical ventilation (MV) is crucial in most cases, although invasive MV, when prolonged, may be associated with increased morbidity and mortality.

## Objective

To verify the severity of illness in ICU patients, at admission, and its relation to invasive MV. To identify whether length of stay on MV or the moment of support implementation (at admission or during the ICU stay) relates to severity of illness and in-hospital/ICU outcomes.

## Methods

Retrospective study, with clinical and surgical adult patients, admitted to the General Hospital of Fortaleza's ICU, from November 2014 to February 2015. Patients were divided into two groups: G1, patients on MV; and G2, nonventilated patients. We analyzed the length of stay on MV (stratified into ranges of 1-7, 8-21 and >21 days) and its relation with disease severity, length of ICU stay and outcomes. Descriptive statistical analysis was used for demographic characters, *t *test for evaluation of continuous variables, chi-square test for categorical variables and ANOVA for multiple comparisons, all with SPSS software. Patients with incomplete data were excluded from the analysis.

## Results

We studied 86 patients, 51.16 % were men, age average was 53.95 ± 19.99 years, average APACHE II score was 14.48 ± 7.21, average admission SOFA score was 4.92 ± 4.01 and average ICU stay period was 13.52 ± 12.88 days. In G1, 62.8 % of patients (*n*= 61 (70.9 %)) had invasive MV when admitted to the ICU. G1 patients had higher severity of illness scores: APACHE II 17.18 vs. 8.85, admission SOFA 6.29 vs. 2.15 (*p*<0.001). Despite stratification of time on MV, we observed no significant difference between the severity scores at admission. ICU and in-hospital mortality was higher in G1 (41.4% vs. 3.6%, p = 0.001, and 52.9 vs. 14.8; p = 0.002, respectively). After the eighth day of MV, the average ICU stay increased in parallel with the duration of MC (p < 0.001). It's relevant to mention that 18.03£ of G1 used MV for more than 2 days. The moment of MC support initiation did not show statistically significant association with severity scores, length of ICU stay, or hospital/ICU mortality.

## Conclusion

MV use was related to severity of illness, length of ICU stay, and in-hospital/ICU mortality. ICU length of stay increased with the duration of MV, from the second week forward. The moment of MV support initial use, however, did not seem relevant.

